# Habitat- and Stage-Associated Variation in Food Resources, Foraging-Attempt Rates, and Behavioral Time Allocation of Wintering Black-Necked Cranes in Linzhou County, XiZang

**DOI:** 10.3390/ani16182923

**Published:** 2026-09-17

**Authors:** Huirui Xing, Bo Yu, Minghang Hu, Zhen Xu, Lingyu Zhai, Zihan Chen, Yuemin Wu, Zhongbin Wang

**Affiliations:** College of Forestry and Grassland, XiZang Agricultural and Animal Husbandry University, No. 100, Yucai West Road, Bayi District, Nyingchi 860000, China; 15689622232@163.com (H.X.); 18689044549@163.com (B.Y.); hmh2580@sina.com (M.H.); 19118599842@163.com (Z.X.); 18738488868@163.com (L.Z.); cchhen0213@163.com (Z.C.); 18368810807@163.com (Y.W.)

**Keywords:** *Black-necked crane*, foraging strategy, potentially available food dry mass, foraging-attempt rate, activity budget, agro-wetland mosaic

## Abstract

Winter food conditions can shape behavioral investment, yet measured food mass and short-term foraging behavior need not covary. We quantified the potentially available food dry mass (FD), foraging-attempt rate (FAR), and 15 min behavioral time allocation of wintering black-necked cranes (*Grus nigricollis* Przevalski, 1876) in five habitats across three sampling stages in Linzhou County, XiZang. Food was sampled in eight independent plots per stage × habitat combination, and 600 valid focal observations were analyzed under a working-independence assumption. Habitat, sampling stage, and their interaction were associated with both FD and FAR (all nominal *p* < 0.001 in the fixed-effects models). Unplowed farmland consistently had the highest FD and FAR, whereas riverbank and shallow-water areas generally had the lowest values. Foraging accounted for 59.2–75.3% of observed time. Because different plots and partly different valleys were sampled among stages, these stage-associated contrasts combine temporal and spatial variation. Plot-level FD–FAR relationships varied among habitats and stages, showing that measured dry mass and a 1 min behavioral rate were not interchangeable indicators of food accessibility, foraging success, or energetic payoff. Future work should revisit fixed plots, retain individual–flock–plot–session identifiers, and quantify food identity, burial depth, hydrological conditions, successful ingestion, and detection-corrected habitat use.

## Simple Summary

Wintering black-necked cranes use farmland, grassland, wetlands, riverbanks, and shallow-water areas. We quantified potentially available food dry mass, foraging-attempt rate, and daytime activity budgets across five habitat types and three sampling stages in Linzhou County, XiZang. Unplowed farmland consistently had the greatest food dry mass and the highest foraging-attempt rate, whereas riverbank and shallow-water areas generally had the lowest values. Foraging dominated the daytime activity budget. Because partly different valleys were surveyed among stages, stage-associated contrasts combine temporal and spatial variation and should not be interpreted as within-site seasonal change. Plot-level relationships between measured food dry mass and foraging-attempt rate varied among habitats and sampling stages, indicating that these metrics describe different components of foraging conditions. The study did not estimate crane density, habitat selection, food accessibility, foraging success, or energetic payoff; therefore, management implications are restricted to the measured resource and behavioral responses. Retaining some unplowed stubble fields and maintaining wetland and shallow-water heterogeneity may help sustain complementary potential food resources through winter.

## 1. Introduction

Food resources are the core determinants of energy balance and habitat utilization for large wading birds during the wintering period. Harvest residues from crops such as highland barley and wheat form high-yield food patches, whereas agricultural and environmental factors—such as plowing, winter irrigation, grazing, and freezing—simultaneously alter food abundance, surface exposure, burial depth, and acquisition costs [1,2,3,4]. Black-necked cranes also consume potatoes; graminoid and cyperaceous plants; underground tubers or rhizomes; and invertebrates. Their dietary composition and ecological niche adapt to seasonal and landscape resource fluctuations [4,5,6]. In agro-wetland landscapes, waterbird food selection is generally modulated by the combined effects of resource accessibility, spatial distribution, and human disturbance [3,7]. Consequently, standing food biomass at the plot level, immediate food accessibility, and an individual’s foraging investment are distinct quantities and need not covary.

The black-necked crane (*Grus nigricollis*) is the only crane species that primarily inhabits the high-altitude regions of the Qinghai–XiZang Plateau and its periphery. It is currently listed as “Near Threatened” by the International Union for Conservation of Nature (IUCN) [8]. Recent studies indicate a global population of approximately 17,000; the Yarlung Zangbo River basin remains a major wintering area, where 9337 individuals were recorded in 2022 and 76.58% occurred in Linzhou, Samzhubzê, Namling, and Lhatse [1]. Linzhou County lies within the Lhasa River basin and Pengbo Valley, where farmland, grassland, wetlands, rivers, and shallow-water zones form a closely connected mosaic used for foraging, vigilance, resting, and seasonal movements [1,9,10].

Behavioral time allocation provides a direct metric for linking resource conditions with individual energetic strategies. Studies on black-necked cranes and other crane species reveal that foraging typically accounts for the largest proportion of diurnal activity; however, investments in foraging, vigilance, resting, and locomotion are adjusted according to season, age, habitat, flock structure, and disturbance levels [11,12,13,14,15]. Crane studies in the Yangtze River basin further indicate that food density, burial depth, substrate penetrability, water levels, and human interference collectively alter foraging-attempt frequencies, handling times, success rates, and the vigilance–foraging trade-off [16,17,18,19,20]. Nevertheless, in the agro-wetland mosaic landscapes of the Qinghai–XiZang Plateau, studies that synchronously quantify food dry mass, foraging-attempt rate, and comprehensive behavioral budgets—and examine their plot-level relationships—remain scarce.

To address this gap, we compared the potentially available food dry mass (FD) and foraging-attempt rate (FAR), together with descriptive 15 min behavioral time allocation, across five foraging habitats and three sampling stages. Specifically, we asked whether (1) FD and FAR were associated with habitat, sampling stage, and their interaction and (2) plot-level FD–FAR associations were consistent across habitat × stage combinations. We expected unplowed farmland (UF) to provide the greatest exposed food mass and support the highest FAR. By measuring resource mass and foraging-attempt intensity within the same balanced design, this study tests whether measured FD covaries with short-term foraging behavior in a high-altitude agro-wetland mosaic.

## 2. Materials and Methods

### 2.1. Study Area

**Figure 1:**
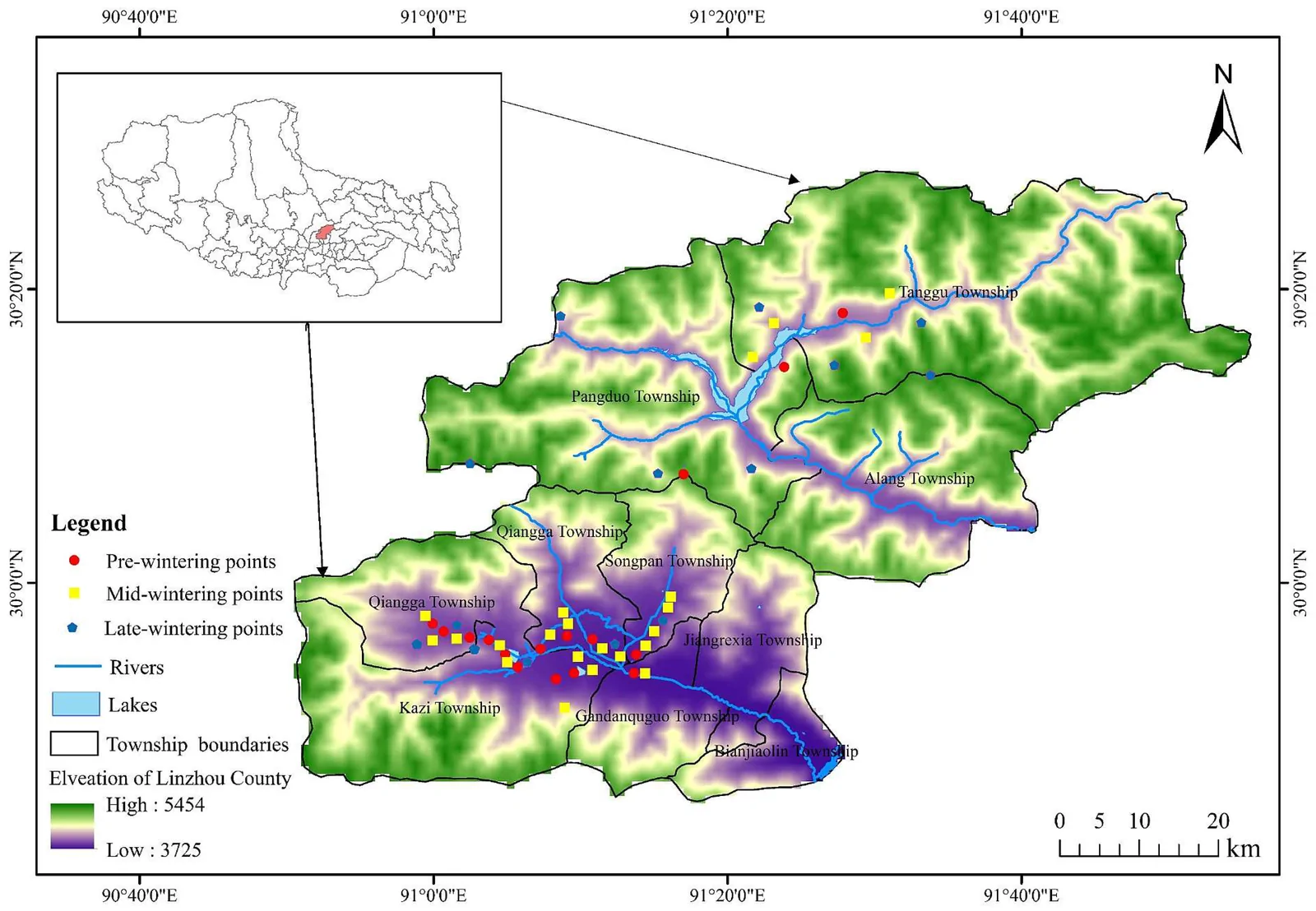
Study area and spatially thinned black-necked crane occurrence locations in Linzhou County, XiZang, China. Red circles, yellow squares, and blue pentagons denote early-, middle-, and late-stage occurrence locations, respectively. The elevation legend gives the minimum and maximum elevation of the displayed raster (3725–5454 m above sea level), not the mean elevation. The 52 locations comprise 49 field records and 3 GBIF records and indicate survey coverage only.

The study was conducted in Linzhou County, Lhasa City, XiZang Autonomous Region, China (approximately 90°51′–91°28′ E, 29°45′–30°08′ N; mean elevation approximately 3700 m; Figure 1). Linzhou lies in the Lhasa River basin, a tributary system of the Yarlung Zangbo River. The main survey area encompassed the agricultural and wetland mosaic of the Pengbo Valley and adjacent northern and eastern tributary valleys, including the Pangduo, Tanggu, and Alang townships. Farmland, grassland, wetlands, riverbanks, and shallow-water areas occur in close proximity within these valleys [1,9,10].

Figure 1 shows 52 spatially thinned crane-occurrence locations (49 field records and 3 Global Biodiversity Information Facility (GBIF) records) used to depict the geographic coverage of the survey. These locations are not food-sampling plots, fixed focal-observation stations, or density estimates. Early- and middle-stage field records were concentrated in the southern Pengbo Valley, whereas late-stage survey coverage also included northern and eastern valleys. We therefore refer to a change in survey coverage, not an expansion of crane distribution.

### 2.2. Survey Design and Sampling

#### 2.2.1. Transect Survey and Plot Setup

Twelve fixed road transects totaling 264 km were surveyed once in each of the early, middle, and late wintering stages (792 km cumulative distance). Road-based transects were used to cover the dispersed valley mosaic efficiently and to locate normally foraging flocks without treating roadside detections as density estimates. A vehicle was driven at 40–60 km h^−1^ through major valleys and associated farmland, grassland, and wetland mosaics. Surveys were conducted in suitable weather within two daylight windows, 08:30–13:00 and 14:00–19:30 China Standard Time; the relatively late upper limit reflects the approximately 2 h solar-time offset at 91° E, and work ended earlier whenever light was insufficient. When a normally foraging flock was located, observers stopped at a non-disruptive distance, recorded the occurrence location and habitat, and selected focal birds. For map preparation, the 177 raw occurrence records (162 field and 15 GBIF records) were spatially thinned after fieldwork in ArcGIS 10.8.1 by retaining one record per 1 km × 1 km grid cell. This produced 52 mapped locations (49 field and 3 GBIF records). No prospective minimum distance was imposed between raw occurrence records because the survey followed encountered flocks; the thinning step was used only to reduce spatial clustering in the coverage map. These records do not represent density or habitat-selection estimates because detection probability and habitat availability were not quantified.

Fieldwork was conducted during 20–26 October 2025 (early stage), 13–20 December 2025 (middle stage), and 18–24 March 2026 (late stage). A balanced design of eight independent macroplots per stage × habitat combination yielded 120 plot-stage units and allowed habitat and stage associations to be compared with equal replication. Different plots were used in the three stages; consequently, stage contrasts are between-group comparisons rather than repeated measurements of the same plots. In addition, early- and middle-stage sampling was concentrated in the southern Pengbo Valley, whereas late-stage coverage also included northern and eastern valleys. Stage-associated differences therefore combine temporal and geographic variation. Plowed-farmland plots met the PF definition at sampling, and individual fields were not followed through a tillage event.

#### 2.2.2. Food Resource Sampling and Dry Mass Determination

Within each macroplot, five 50 cm × 50 cm quadrats were positioned in a quincunx to distribute subsamples across the plot and were excavated to a depth of 10 cm. Potential food items were collected on the basis of direct feeding observations, foraging traces, and published diet information, and included identifiable grain, underground tubers or rhizomes, edible herbaceous material, and invertebrates. The five quadrats were pooled as one composite sample (total area 1.25 m^2^) so that the macroplot, rather than each quadrat, remained the independent sampling unit. After sand, silt, and non-food material were removed, samples were dried at 65 °C for 8–12 h and reweighed at 15 min intervals until constant mass, thereby standardizing measurements for residual moisture.

Potentially available food dry mass per unit area was calculated as

FD (g m^−2^) = dry mass of the five-quadrat composite sample (g)/1.25 m^2^.

#### 2.2.3. Focal Animal Observation

For each stage × habitat combination, 40 normally foraging black-necked cranes were selected as focal subjects. Continuous focal sampling was chosen to link individual food-directed movements with a complete activity sequence. Each bird was observed for 1 min to quantify the frequent bill movements used to calculate FAR and then for 15 min to record its activity budget while limiting loss of the unmarked focal subject. Observations were distributed across the two daylight periods defined above. Of the 652 attempted observations, 52 were excluded because the bird became obscured or departed before completion, leaving 600 valid records (5 habitats × 3 stages × 40 observations).

A foraging attempt was defined as a discrete, food-directed peck, probe, or digging movement of the bill. FAR was calculated as the number of attempts divided by the effective observation time:

FAR (attempts min^−1^) = number of foraging attempts/effective observation duration (min).

To reduce, but not eliminate, pseudoreplication, focal birds were preferentially selected from different flocks, dates, or observation periods, and generally no more than 1–3 individuals were sampled from one flock within a given period. Individual identity could not be confirmed in unmarked birds, and complete flock and session identifiers were not available in the analytical dataset used for this retrospective revision. The full sampling hierarchy therefore could not be reconstructed. Repeated sampling of the same crane and residual dependence among birds sharing a flock or location therefore cannot be excluded. FAR results are therefore interpreted under a working-independence assumption rather than treating the 600 focal records as guaranteed independent biological replicates.

Three to five trained observers participated in fieldwork. For a retrospective reliability assessment, a designated pair independently recorded the same focal bird during 60 matched 15 min sessions, with four sessions from each of the 15 stage × habitat combinations. Both observers used the same ethogram and recording protocol. The paired records were used only for reliability analysis and were not counted as additional observations in the ecological analyses.

Observers remained approximately 0.5–1.5 km from focal flocks and worked from a concealed low-profile position or a stationary vehicle. Each site visit lasted approximately 0.5–3 h. No quantitative index of traffic, people, livestock, dogs, or other disturbance was collected; consequently, the study does not test effects of human disturbance.

### 2.3. Habitat, Behavior, and Wintering-Stage Definitions

The wintering season was divided into early (late October to mid-November), middle (mid-November to late February), and late (early March to late April) stages. The field sampling dates within these periods are given in Section 2.2.1.

Habitats were defined at the time of sampling as follows: UF, post-harvest farmland that had not been plowed and retained surface crop residues and scattered grain; PF, post-harvest farmland that had already been plowed, with residues partly or fully covered by soil; GL, natural or semi-natural herbaceous vegetation; WL, permanently or seasonally wet marsh meadow or hydrophytic vegetation; and RSW, riverbanks, exposed mudflats, and shallow-water areas. Crop identity, harvest date, and exact tillage date were not recorded for each plot.

Following focal animal sampling principles [11,12,13,14,15,21,22], six mutually exclusive behaviors were recorded second by second during each 15 min session: foraging (FO; searching, food-directed pecking or probing, digging, swallowing, and food handling); vigilance (VI; head raised and neck extended while scanning); resting/maintenance (RM; stationary resting and preening); locomotion (LO; walking, running, or other non-foraging displacement); flight (FL); and aggression (AG; threats, chasing, or physical conflict). The six durations summed to 900 s for each valid observation.

Behavioral proportion (%) = behavior duration (s)/900 s × 100.

### 2.4. Data Processing and Statistical Analysis

The macroplot was the independent unit for FD (*n* = 120; eight plots per stage × habitat combination), whereas the focal observation was the nominal analysis unit for FAR (*n* = 600; 40 observations per combination). The five food quadrats within a macroplot were pooled and were not treated as independent replicates. Selecting birds from different flocks, dates, or periods and limiting sampling to generally 1–3 birds per flock reduced clustering but did not guarantee independence. Because complete individual, flock, and session identifiers were unavailable, the full individual–flock–plot–session hierarchy could not be reconstructed retrospectively. The FAR model therefore uses a working-independence assumption, and its nominal *p*-values may understate uncertainty arising from residual clustering.

Separate two-factor fixed-effects models evaluated associations of habitat, sampling stage, and their interaction with FD and FAR. Stage was treated as a between-group factor because different plots were sampled at each stage. The FAR model was retained as a transparent working-independence analysis and is interpreted cautiously in view of the clustering limitation described above. Diagnostics included Q–Q and residual-versus-fitted plots, Shapiro–Wilk tests of residual normality, and mean-centered Levene tests across the 15 stage × habitat cells. For FD, W = 0.979, *p* = 0.053, and Levene F(14, 105) = 2.321, *p* = 0.008. For FAR, W = 0.998, *p* = 0.869, and Levene F(14, 585) = 1.054, *p* = 0.397. Because FD variances were heterogeneous, the model was repeated after square-root transformation; assumptions were then supported (W = 0.979, *p* = 0.064; Levene F(14, 105) = 1.280, *p* = 0.232). Untransformed values were retained for interpretation.

Significant interactions were followed by simple-effects comparisons among habitats within each stage and among stages within each habitat, with Tukey’s HSD adjustment. Figure captions define the letter groups and asterisk thresholds used for these comparisons.

Because the six behavioral proportions sum to 1, they were treated as compositional [22] and are reported only as descriptive mean percentages in Supplementary Figure S1. Pooled seconds were not analyzed as independent observations, and no inferential comparison of behavioral composition was made.

Inter-observer absolute agreement for the six behavioral durations and FAR was evaluated with a two-way random-effects, single-measure ICC [ICC(2,1)] [23]. Ninety-five percent confidence intervals were obtained from 20,000 paired-session bootstrap resamples. The Bland–Altman mean bias (Observer 2 minus Observer 1) was examined as an additional diagnostic [24].

For exploratory plot-level regressions, FAR was averaged across the five focal observations associated with each macroplot and paired with that plot’s FD. Fifteen separate regressions (five habitats × three sampling stages) were fitted, each using eight macroplots (*n* = 8). Thus, each regression used one macroplot as the analysis unit; the analysis did not pool 24 records per habitat. Slopes and R^2^ values are interpreted only as descriptive trends.

Analyses were conducted in IBM SPSS Statistics 26.0 (IBM Corp., Armonk, NY, USA) and R 4.3.0 [25], with ggplot2 used for figures [26]; ArcGIS 10.8.1 (Esri, Redlands, CA, USA) was used for mapping and spatial thinning. Unless stated otherwise, values are mean ± standard deviation, tests are two-tailed, and α = 0.05.

## 3. Results

### 3.1. Inter-Observer Reliability

All 60 paired sessions passed the matching and integrity checks. ICC (2,1) estimates indicated high absolute agreement and ranged from 0.952 to 0.997. Estimates (95% confidence interval) were 0.997 (0.994–0.998) for FO; 0.952 (0.923–0.972) for VI; 0.995 (0.992–0.997) for RM; 0.981 (0.963–0.991) for LO; 0.981 (0.962–0.992) for FL; 0.974 (0.952–0.987) for AG; and 0.971 (0.935–0.989) for FAR. Mean Observer 2 minus Observer 1 differences were small and showed no consistent direction across measures.

### 3.2. Behavioral Time Allocation

Foraging was the largest component of the daytime activity budget in all 15 stage × habitat combinations, accounting for 59.2–75.3% of observed time (Supplementary Figure S1). These percentages are descriptive; no inferential differences in behavioral composition among stages or habitats were tested.

### 3.3. Habitat- and Stage-Associated Variation in Food Resources

**Figure 2:**
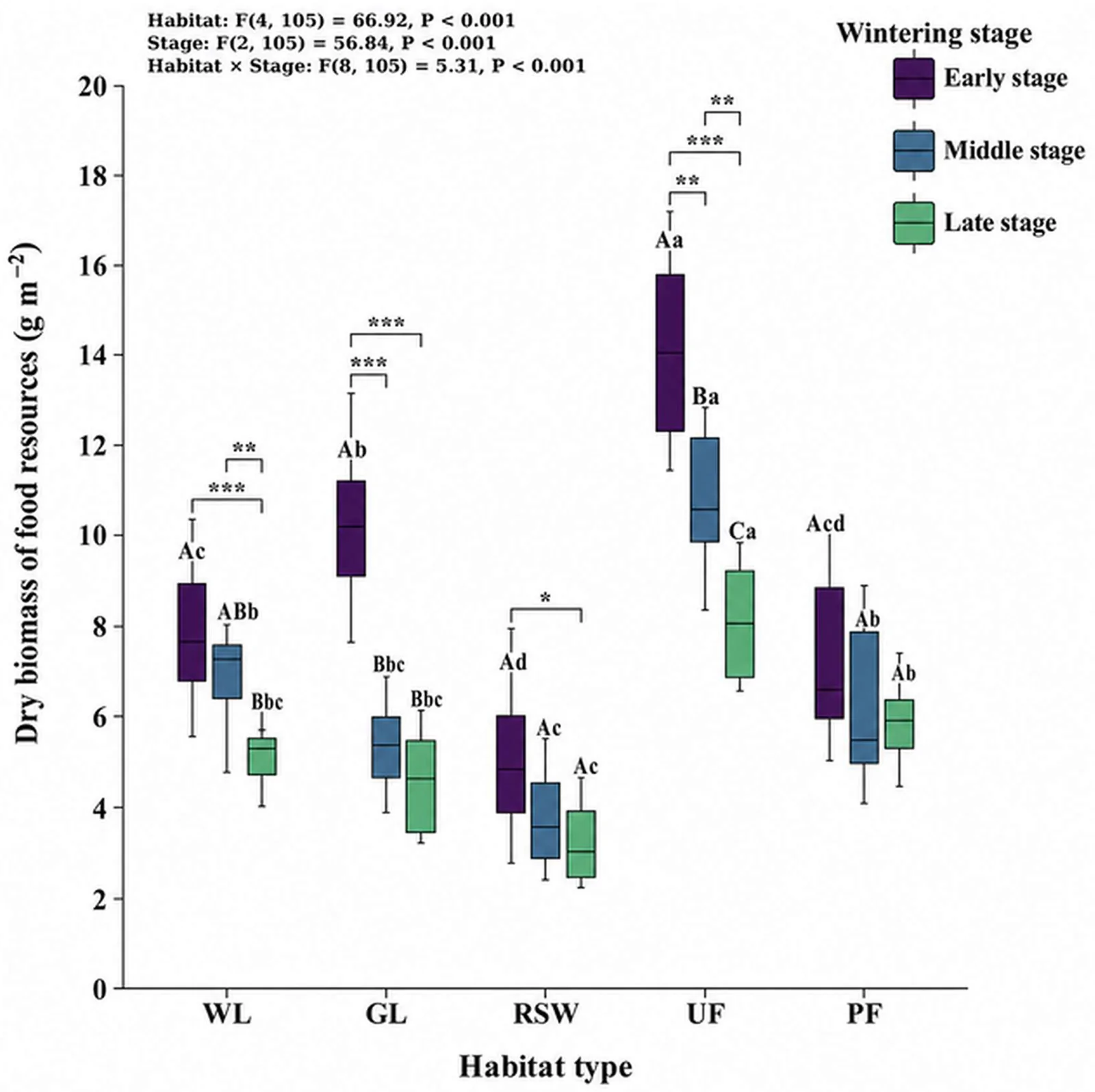
Potentially available food dry mass across five habitats and three sampling stages. Boxes show interquartile ranges, center lines show medians, and whiskers show observed ranges. Purple, blue, and green boxes represent the early, middle, and late wintering stages, respectively. Uppercase letters compare stages within a habitat, whereas lowercase letters compare habitats within a stage; groups without a shared letter differ significantly. Brackets show Tukey-adjusted pairwise comparisons: * *p* < 0.05, ** *p* < 0.01, *** *p* < 0.001. Habitats are ordered WL, GL, RSW, UF, and PF. UF, unplowed farmland; PF, plowed farmland; GL, grassland; WL, wetland; RSW, riverbank and shallow-water areas.

Under the fixed-effects model, FD was associated with habitat [F(4, 105) = 66.92, *p* < 0.001], sampling stage [F(2, 105) = 56.84, *p* < 0.001], and their interaction [F(8, 105) = 5.31, *p* < 0.001] (Figure 2). Habitat rankings were UF > PF > GL > WL > RSW in the early stage; UF > GL > WL > PF > RSW in the middle stage; and UF > WL > GL > PF > RSW in the late stage. Square-root-transformed FD gave the same conclusions (habitat: F(4, 105) = 64.27; stage: F(2, 105) = 51.96; interaction: F(8, 105) = 3.80; all *p* < 0.001).

Stage-specific mean FD in UF was 14.20 ± 2.26 g m^−2^ in the early stage; 10.80 ± 1.59 g m^−2^ in the middle stage; and 8.10 ± 1.29 g m^−2^ in the late stage. Corresponding values were 10.30 ± 1.95, 5.40 ± 1.04, and 4.60 ± 1.21 g m^−2^ in PF; 7.90 ± 1.65, 6.90 ± 1.25, and 5.10 ± 0.60 g m^−2^ in GL; 7.20 ± 1.94, 6.20 ± 1.81, and 5.90 ± 1.00 g m^−2^ in WL; and 5.10 ± 1.78, 3.80 ± 1.26, and 3.30 ± 0.99 g m^−2^ in RSW. Because different plots and partly different valleys were sampled among stages, these values describe stage-associated between-plot contrasts that combine temporal and spatial variation; they do not represent depletion measured repeatedly in the same fields.

### 3.4. Habitat- and Stage-Associated Variation in Foraging-Attempt Rate

**Figure 3:**
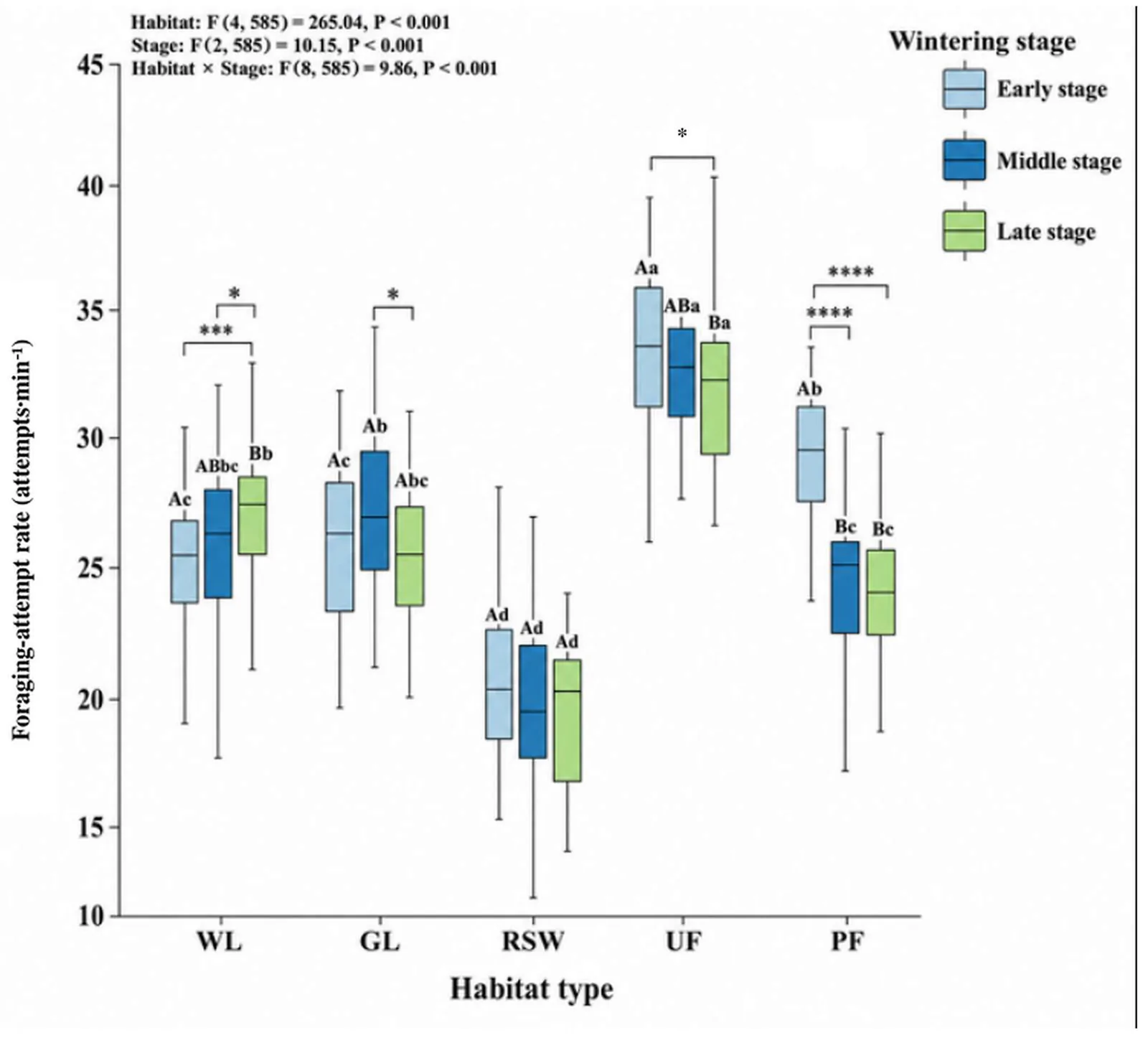
Foraging-attempt rate of black-necked cranes across five habitats and three sampling stages. Boxes show interquartile ranges, center lines show medians, and whiskers show observed ranges. Light blue, dark blue, and green boxes represent the early, middle, and late wintering stages, respectively. Uppercase letters compare stages within a habitat, whereas lowercase letters compare habitats within a stage; groups without a shared letter differ significantly. Brackets show Tukey-adjusted pairwise comparisons: * *p* < 0.05, *** *p* < 0.001, and **** *p* < 0.0001. FAR is the number of food-directed attempts per minute and does not measure ingestion success. Habitats are ordered WL, GL, RSW, UF, and PF. UF, unplowed farmland; PF, plowed farmland; GL, grassland; WL, wetland; RSW, riverbank and shallow-water areas.

Under the working-independence fixed-effects model, FAR was associated with habitat [F(4, 585) = 265.04, *p* < 0.001], sampling stage [F(2, 585) = 10.15, *p* < 0.001], and their interaction [F(8, 585) = 9.86, *p* < 0.001] (Figure 3). Habitat rankings matched those for FD: UF > PF > GL > WL > RSW in the early stage; UF > GL > WL > PF > RSW in the middle stage; and UF > WL > GL > PF > RSW in the late stage. Because residual within-flock or within-location clustering cannot be excluded, the nominal *p*-values should be interpreted cautiously.

UF had the highest FAR across stages (approximately 31.8–33.5 attempts min^−1^), whereas RSW had the lowest (approximately 19.5–20.8 attempts min^−1^). PF was higher in the early stage than in the middle and late stages, whereas WL was higher in the late stage than in the earlier stages.

### 3.5. Plot-Level Relationship Between Food Dry Mass and Foraging-Attempt Rate

**Figure 4:**
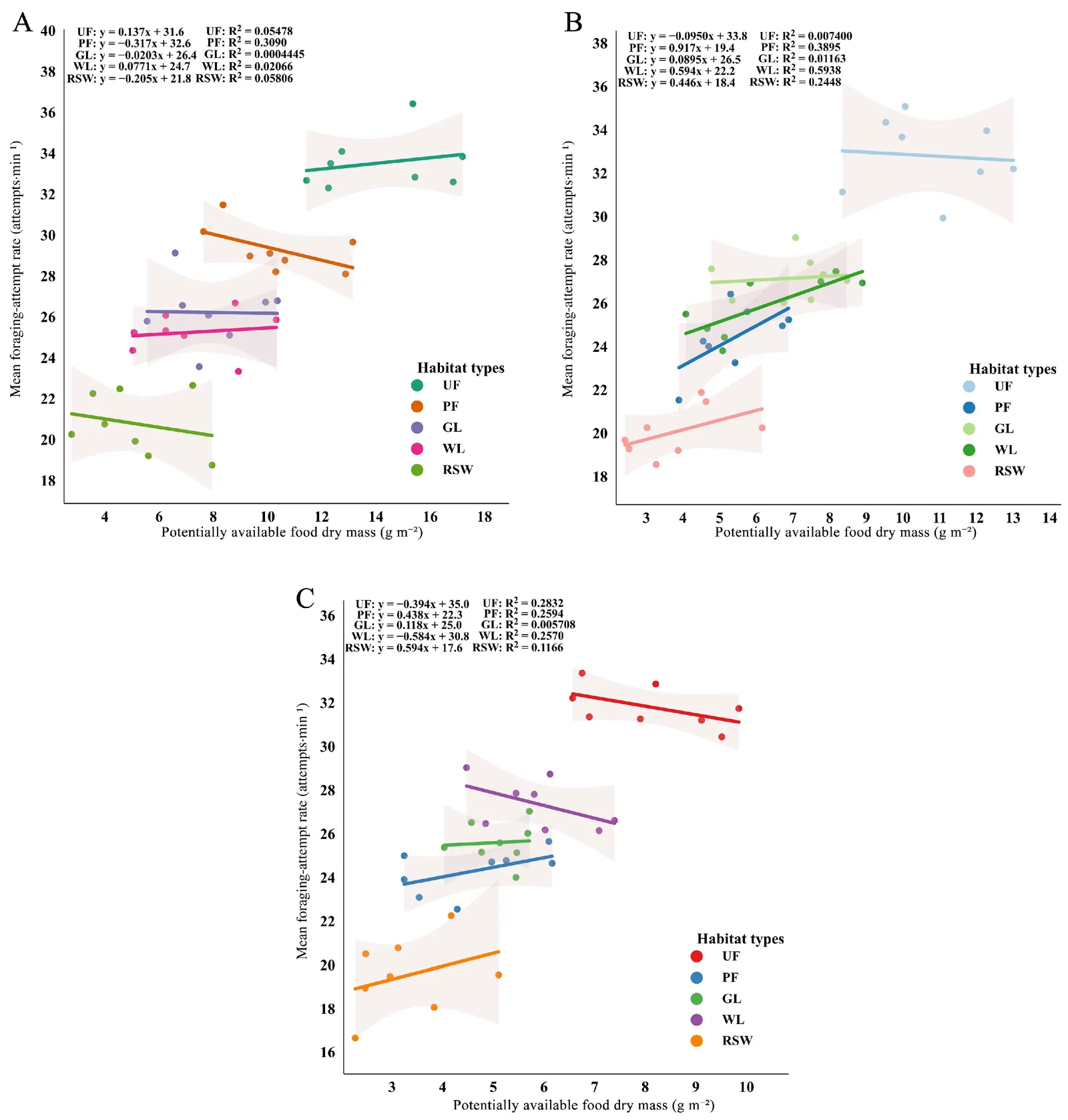
Exploratory plot-level relationships between potentially available food dry mass and mean foraging-attempt rate. Panels (**A**–**C**) show the early, middle, and late sampling stages, respectively. Each point is one macroplot, paired with the mean FAR of its five focal observations; colored solid lines are ordinary least-squares fits and shaded bands are 95% confidence intervals. Fifteen habitat × stage regressions were fitted, and each fit used eight macroplots (*n* = 8). Equations and R^2^ values printed in each panel describe exploratory fitted trends only. Panel letters identify sampling stages and are not significance-group labels; colors correspond to the habitat codes in the legend. UF, unplowed farmland; PF, plowed farmland; GL, grassland; WL, wetland; RSW, riverbank and shallow-water areas.

Across the 15 exploratory regressions, plot-level relationships between FD and FAR varied among habitats and sampling stages (Figure 4). In the early stage, all relationships were weak and PF showed the largest absolute trend (negative; R^2^ = 0.3090). In the middle stage, the strongest positive relationship occurred in WL (R^2^ = 0.5938), with weaker positive trends in PF (R^2^ = 0.3895) and RSW (R^2^ = 0.2448). In the late stage, UF and WL showed negative trends, PF and RSW showed positive trends, and GL was nearly flat. These regressions are exploratory and are not used as the principal basis for biological inference.

Each fitted line was based on eight macroplots within one habitat × stage combination. The analysis unit was therefore one macroplot, not 24 pooled observations per habitat. These regressions are descriptive and do not establish causal relationships among food dry mass, unmeasured accessibility, habitat characteristics, and individual behavior.

## 4. Discussion

### 4.1. Scope and Limitations of Inference

The main strengths of this study are the parallel measurements of FD, FAR, and behavioral time allocation across a replicated habitat × stage design. Five limitations define the appropriate scope of inference. First, line-transect occurrence records were used to locate flocks and describe survey coverage; detection probability and habitat availability were not estimated, so the data cannot quantify crane density or habitat selection. Second, different macroplots and partly different valleys were sampled among stages; stage-associated contrasts therefore combine temporal and spatial variation and cannot be interpreted as within-field depletion. Third, focal birds were unmarked and complete flock and session identifiers were unavailable. The individual–flock–plot–session hierarchy could not be reconstructed, so repeated individuals and dependence within flocks or locations cannot be excluded; the fixed-effects FAR model may consequently understate uncertainty. Fourth, pooled food samples did not distinguish food type, particle size, energetic value, burial depth, substrate hardness, or water depth. Fifth, FAR counts food-directed attempts, not successful ingestions or energy intake. These limitations prevent causal interpretations of the fixed-effects and exploratory regression results.

### 4.2. Food Resources and Agricultural Condition

UF had the highest FD in all three stages and also supported the highest FAR. This agreement is consistent with greater surface exposure of post-harvest grain in unplowed fields and with previous evidence that agricultural residues are important winter food for cranes [1,2,3,4,6]. PF plots had already been plowed when sampled; therefore, the lower middle- and late-stage values cannot be attributed to a tillage event observed during this study. They may reflect differences among independently sampled fields, prior burial of residues, consumption by animals, or other unmeasured agricultural conditions. Crop identity, harvest timing, and tillage timing should be recorded plot by plot in future work to separate these mechanisms.

The stage-associated difference in measured FD was smaller in WL than in most other habitats from the middle to late sampling stages, whereas RSW consistently had low measured FD. The composite dry-mass metric did not measure food accessibility. Foods in wetlands and shallow water may occur below water or within sediment and may require longer probing, searching, or handling, potentially reducing attempts per minute even when food is present [16,17,18]. These are plausible explanations rather than measured mechanisms. Thus, the results identify contrasts in measured resource mass, not the availability, accessibility, energetic value, or functional quality of each habitat. The comparison shows that high farmland residue mass and the smaller stage-associated change in wetland FD represent different resource patterns within the sampled alpine agro-wetland mosaic.

### 4.3. Foraging-Attempt Rate and Potential Food Dry Mass

The broad habitat rankings of FAR and FD were similar, most clearly because UF was highest and RSW lowest. However, the plot-level association was not uniform. The strongest positive trend occurred in middle-stage WL, with weaker positive trends in PF and RSW, whereas several other habitat × stage combinations showed little or negative association. This pattern indicates that dry mass and a 1 min behavioral rate describe different components of foraging conditions. Potential moderators include the mixture of food types in pooled samples, whether food was exposed or buried, substrate and water conditions, spatial aggregation, and handling time. Because these variables were not measured directly, the proposed mechanisms remain hypotheses.

FAR should not be equated with foraging success or energetic payoff. A peck, probe, or digging movement may end in ingestion or failure, and foods of different sizes can generate different attempt rates for the same energy return. A bird making more attempts per minute may be exploiting abundant food efficiently or compensating for difficult access. The present data therefore support comparisons of attempt intensity only. Video-based scoring of successful ingestion, food identity, handling time, and food mass per successful event would be needed to estimate intake rate or energetic profitability [18,19]. The principal result is the scale-dependent FD–FAR relationship: broad habitat rankings were similar even though plot-level associations were inconsistent. Measured dry mass should therefore not be used as a complete surrogate for immediate foraging conditions because accessibility was not quantified.

### 4.4. Conservation Implications

The strongest management implication follows directly from the measured FD and FAR patterns: retaining some unplowed, residue-bearing fields through winter could preserve surface residues as potential food resources. Because the study did not estimate density or habitat selection, this recommendation concerns resource provision rather than demonstrated numerical preference. Management should also maintain a heterogeneous mosaic of wetlands, mudflats, and shallow-water areas, whose measured food resources and other functions may complement farmland [17,27,28,29,30]. Implementation should be coordinated with farmers and evaluated through monitoring of crop type, harvest and tillage dates, food burial, water depth, crane abundance, and standardized disturbance metrics.

Future work should repeat sampling over multiple winters, revisit fixed plots, retain stable individual, flock, plot, and session identifiers for hierarchical mixed-effects analyses, estimate habitat-specific crane density with detection-aware methods, and distinguish successful ingestion from attempts. Linking standardized density or use–availability data to resource, hydrological, and agricultural measurements would directly test whether crane abundance shifts from unplowed fields toward wetlands or shallow-water areas as winter progresses.

## 5. Conclusions

Using a balanced habitat × stage design comprising 120 independent macroplots and 600 valid focal observations, this study found habitat- and stage-associated variation in potentially available food dry mass (FD) and foraging-attempt rate (FAR) in fixed-effects analyses (all nominal *p* < 0.001). Because different plots and partly different valleys were sampled among stages, these contrasts combine temporal and spatial variation. Unplowed farmland consistently had the highest FD and FAR, whereas riverbank and shallow-water areas generally had the lowest values. Foraging dominated the daytime activity budget, accounting for 59.2–75.3% of observed time. At the habitat-ranking level, FD and FAR were broadly aligned; however, their plot-level relationships varied among habitats and stages. This scale-dependent pattern shows that measured dry mass and a short-term behavioral rate are not interchangeable indicators of food accessibility, foraging success, or energetic payoff. The findings support retaining some unplowed, residue-bearing fields through winter and maintaining a heterogeneous mosaic of wetlands, mudflats, and shallow-water areas. These recommendations concern measured resource provision and behavioral responses rather than demonstrated habitat selection. The working-independence FAR analysis may understate uncertainty because complete individual and flock identifiers were unavailable. Future studies should revisit fixed plots, retain hierarchical identifiers, and jointly measure food identity, burial depth, substrate penetrability, water depth, disturbance, ingestion success, and habitat-specific crane abundance.

## Data Availability

The original contributions presented in this study are included in the article/Supplementary Material. Further inquiries can be directed to the corresponding author(s).

## Supplementary Materials

The following supporting information can be downloaded at https://www.mdpi.com/article/10.3390/ani16182923/s1. Figure S1: Descriptive behavioral time allocation of black-necked cranes across five habitats and three wintering stages. Each stacked bar shows the mean composition of 40 valid 15 min focal observations and sums to 100%. Panels A–C show the early, middle, and late stages, respectively. UF, unplowed farmland; PF, plowed farmland; GL, grassland; WL, wetland; RSW, riverbank and shallow-water areas; FO, foraging; VI, vigilance; RM, resting/maintenance; LO, locomotion; FL, flight; AG, aggression.
