# Supplementary material for: Habitat- and Stage-Associated Variation in Food Resources, Foraging-Attempt Rates, and Behavioral Time Allocation of Wintering Black-Necked Cranes in Linzhou County, XiZang
Source: Animals (Basel). 2026 Sep 17;16(18):2923. doi: 10.3390/ani16182923 (PMC13603172; doi:10.3390/ani16182923)
Supplement: Supplementary file 1 [file animals-16-02923-s001.zip › animals-4491576-supplementary.pdf]

Supplementary Materials

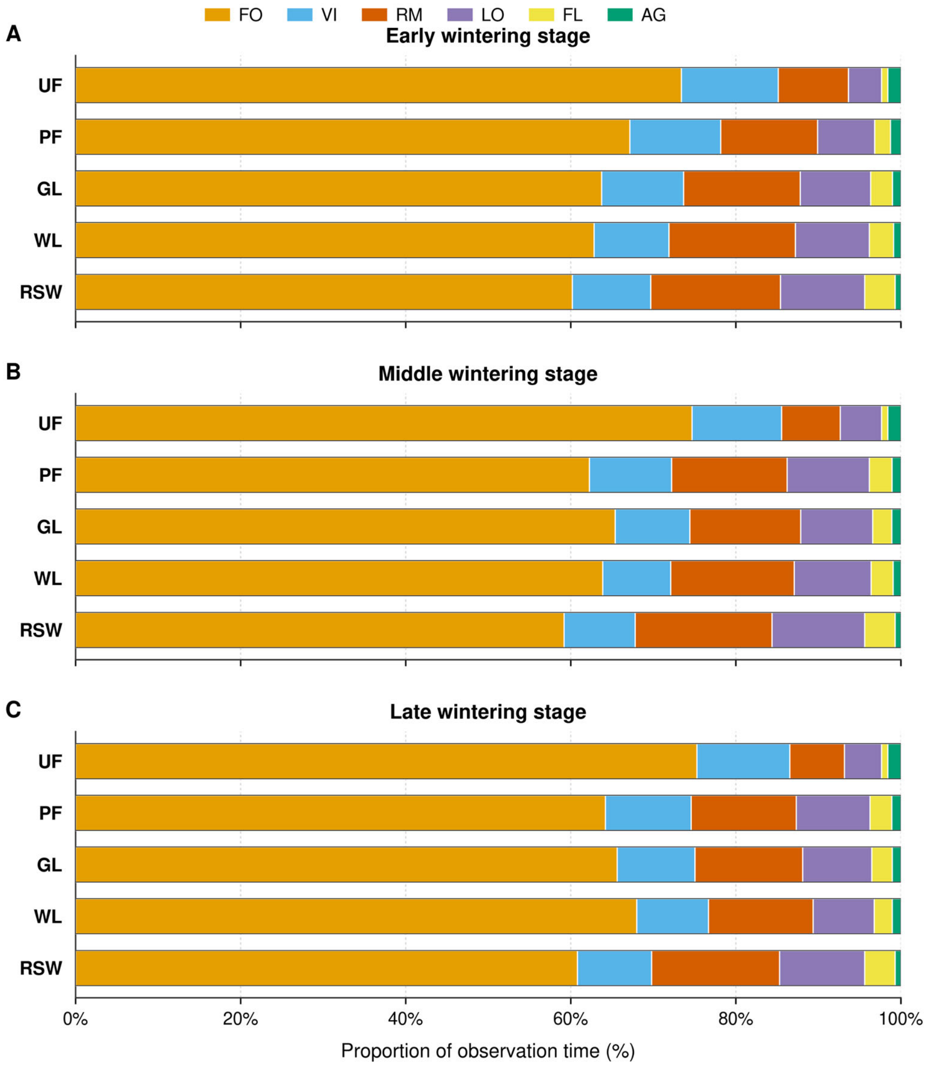

Supplementary Figure S1. Descriptive behavioral time allocation of black-necked cranes across five habitats and three sampling stages. Each stacked bar shows the mean composition of 40 valid 15 min focal observations and sums to 100%; the length of each colored segment is the mean percentage of observation time. Panels (**A**–**C**) show the early, middle, and late sampling stages, respectively. Gold, FO (foraging); blue, VI (vigilance); orange, RM (resting/maintenance); purple, LO (locomotion); yellow, FL (flight); green, AG (aggression). UF, unplowed farmland; PF, plowed farmland; GL, grassland; WL, wetland; RSW, riverbank and shallow-water areas.
